# Interfacial Photothermal Heat Accumulation for Simultaneous Salt Rejection and Freshwater Generation; an Efficient Solar Energy Harvester

**DOI:** 10.3390/nano12183206

**Published:** 2022-09-15

**Authors:** Zhou Wei, Naila Arshad, Chen Hui, Muhammad Sultan Irshad, Naveed Mushtaq, Shahid Hussain, Matiullah Shah, Syed Zohaib Hassan Naqvi, Muhammad Rizwan, Naeem Shahzad, Hongrong Li, Yuzheng Lu, Xianbao Wang

**Affiliations:** 1 Hubei Key Laboratory of Polymer Materials, Collaborative Innovation Center for Advanced Organic Chemical Materials Co-Constructed by the Province and Ministry, Ministry of Education Key Laboratory for the Green Preparation and Application of Functional Materials, School of Materials Science and Engineering, Hubei University, Wuhan 430062, China; 2 Guangdong Provincial Key Laboratory of Micro/Nano Optomechatronics Engineering, College of Mechatronics and Control Engineering, Shenzhen University, Shenzhen 518060, China; 3 Institute of Quantum Optics and Quantum Information, School of Science, Xi’an Jiaotong University (XJTU), Xi’an 710049, China; 4 School of Materials Science and Engineering, Jiangsu University, Zhenjiang 212013, China; 5 Department of Physics, University of Wah, Wah Cantonment 47040, Pakistan; 6 Department of Electronics Engineering, University of Engineering and Technology, Taxila 47080, Pakistan; 7 CE Wing, MCE, National University of Sciences and Technology Risalpur Campus, Risalpur 430062, Pakistan; 8 School of Electronic Engineering, Nanjing Xiaozhuang University, Nanjing 211171, China

**Keywords:** photothermal heat, interfacial, solar evaporation, salt-rejection, water scarcity

## Abstract

Water scarcity has emerged as an intense global threat to humanity and needs prompt attention from the scientific community. Solar-driven interfacial evaporation and seawater desalination are promising strategies to resolve the primitive water shortage issue using renewable resources. However, the fragile solar thermal devices, complex fabricating techniques, and high cost greatly hinder extensive solar energy utilization in remote locations. Herein, we report the facile fabrication of a cost-effective solar-driven interfacial evaporator and seawater desalination system composed of carbon cloth (CC)-wrapped polyurethane foam (CC@PU). The developed solar evaporator had outstanding photo-thermal conversion efficiency (90%) with a high evaporation rate (1.71 kg m^−2^ h^−1^). The interfacial layer of black CC induced multiple incident rays on the surface allowing the excellent solar absorption (92%) and intensifying heat localization (67.37 °C) under 1 kW m^−2^ with spatially defined hydrophilicity to facilitate the easy vapor escape and validate the efficacious evaporation structure using extensive solar energy exploitation for practical application. More importantly, the long-term evaporation experiments with minimum discrepancy under seawater conditions endowed excellent mass change (15.24 kg m^−2^ in consecutive 8 h under 1 kW m^−2^ solar irradiations) and promoted its operational sustainability for multi-media rejection and self-dissolving potential (3.5 g NaCl rejected from CC@PU surface in 210 min). Hence, the low-cost and facile fabrication of CC@PU-based interfacial evaporation structure showcases the potential for enhanced solar-driven interfacial heat accumulation for freshwater production with simultaneous salt rejection.

## 1. Introduction

Freshwater scarcity is recognized as a strategically important global issue that is essential to human survival, as well as economic and social progress [1,2]. Due to population growth, climate change, and the spread of pollution over the last few decades, many people are suffering from freshwater shortages due to excessive natural evaporation caused by global warming [2]. Solar energy is a promising and ample renewable energy source that can potentially meet all the energy crises upon efficient harvesting [3,4]. Recently, solar-thermal energy conversion is attaining extensive attention from the scientific community for many applications, such as power generation, domestic water heating, desalination, and other industrial processes [5,6]. Interfacial solar desalination offers a promising solution to yield freshwater directly out of solar energy without extra energy input, peculiarly urgent for developing countries and remote areas without basic infrastructures [7,8].

To date, significant efforts and several approaches have been invested in order to attain high-efficiency evaporation performance by upgrading several factors i.e., (1) development of low-cost and facile fabricating evaporation devices for maximum solar absorption accompanied by reduced evaporation enthalpy of water [9], (2) optimization of the structural design to prevent salt accumulation, reducing heat losses [10], and complete exploitation of the available heat for vapor generation [11]. Renewable solar energy is a green and pollution-free source for the extensive utilization of desalination applications to get fresh water [5]. Nevertheless, the seawater is occupied by a high salt concentration of approximately 3.5 wt% NaCl which can ultimately accumulate on the evaporation layer and inside water channels of evaporation structures sacrificing the thermal insulation which results in decreased solar absorption and evaporation efficiency during the desalination operation [1,12]. During seawater desalination, several photothermal designs are created from the salt accumulation on the evaporator surface, which not only severely affects the light absorption area but also inevitably blocks steam escape channels during steam generation [3,13]. In 2020, Qin et al. reported the fabrication of a solar water desalination system by drilling a millimeter-scale channel array in a wood substrate with enhanced hydraulic conductivity via concentration gradient to transfer the salt back into the bulk solution, nanofiber-based evaporators to induce salt transfer from the evaporating interface to the water along the holes between nanofibers, and Janus structures to make salt ions stay on the hydrophilic layer and dissolve back into source water without crystallizing on the evaporator surface [14]. However, the excessive conduction of the heat downward via rapid convection and the inevitable passage of a portion of light through the holes of the evaporator decreases the thermal-conversion efficiency of the device. Similarly, in 2018, Xu et al. reported the preparation of a carbon black-decorated polymethylmethacrylate (PMMA@CB) hydrophobic layer to prevent the salt accumulation between PMMA@CB and the hydrophilic water transport layer (polyacrylonitrile) [15]. Indeed, even with advanced evaporation structures with the optimization of several factors, there remain some key challenges to further enhance the photothermal conversion efficiency of the resulting devices [15]. More significantly, the thermal management, heat losses, substrate selection, water transport, and salt barrier should all be tuned at the same time to establish a stable and long-term self-desalinating solar-driven evaporation system [6].

Carbon-based nanomaterials have been reported extensively for outstanding photostability and broad-band optical absorption, low cost, and environmentally friendly nature for the preservation of surroundings [1,16]. Various carbon-based nanomaterials have been developed successfully for efficient photothermal conversion and steam generation i.e., hydrophilic carbon foam, hydrogels, aerogels, carbon-nanotubes (CNT’s)-based modified structures, and biomass-derived carbon composites [17,18]. However, challenges are still being faced in terms of accessibility to a stable and flexible material structure, low cost, facile fabrication, and accumulation of salt on the photothermal surface which severely reduces its potential for practical application [19]. Carbon cloth (CC), made from organic polymer, is a good solar light absorber and has been used in many photothermal conversion applications. CC offers broad-band optical absorption, high tensile strength, low thermal conductivity, flexibility, and high tensile strength with low density, providing it with optimum conditions for efficient solar steam generation applications [20]. More importantly, the low thermal conductivity of the carbon cloth enables excellent thermal localization on the top surface with less thermal dissipation across the fabric area assisting in the excellent thermal accumulation on the photothermal layer during steam generation [20]. The highly porous structure of CC facilities water transport via capillary force, which constitutes a continuous channel for the water molecules to add up at the air–water interface. CC also owes excellent thermal stability at high temperatures, which endorses its exceptional potential for practical application in remote areas [21].

**Figure 1:**
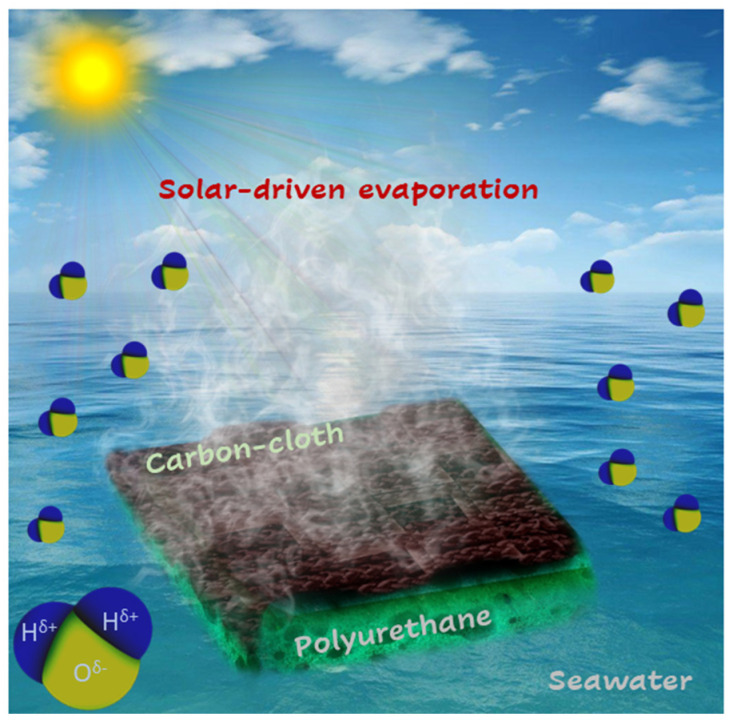
Schematic illustration of interfacial photothermal heat accumulation inspired solar evaporator derived from carbon-cloth-wrapped polyurethane foam for continuous salt-resistance and steam generation.

Herein, based on the excellent photothermal conversion potential and high thermal stability of CC, we report the facile fabrication of a solar-driven interfacial evaporation system composed of carbon-cloth-wrapped polyurethane foam (CC@PU) for clean water generation and seawater desalination, as illustrated in Figure 1. The CC@PU solar evaporator shows excellent light absorption (92%), excellent thermal management, and a high evaporation rate (1.71 kg m^−2^ h^−1^) under one solar intensity. The incorporation of hydrophilic PU foam with numerous open pores’ assembly endorsers a quick water supply with simultaneous thermal restriction to the downward matrix as the thermal conductivity of PU is even lower than CC, while the less thermal conduction of CC enables the thermal localization on the incident side of CC with no thermal passage on other side and achieves a surface temperature enhancement up to 67.37 °C under 1 kW m^−2^ providing it with highly desired conditions to build a highly efficient interfacial solar evaporator. Moreover, the solar evaporator shows excellent regenerating potential by redissolving the salt back into bulk water through the highly porous structure with excellent stability (15.24 kg m^−2^ mass change of water in consecutive 8 h operation under 1 kW m^−2^ solar irradiance) using seawater conditions. Furthermore, a slope-shaped glass condenser demonstrates the practical application of facile development of solar evaporation setup. Hence, the low-cost, facile-fabricated CC@PU-based interfacial solar evaporator shows promising potential for practical application in remote areas and opens up tremendous opportunities in solar water evaporation technology.

## 2. Materials and Methods

### 2.1. Materials

Carbon cloth (CC, W0S1009) was utilized as a photothermal layer acquired from CeTech. Co., Ltd. Kowloon, Hong Kong. The hydrophilic Polyurethane foam (PU) substrate was bought from Zhong Sheng Trade Co. Wuyi, Zhejiang, P.R. China. Sodium Chloride (NaCl) was used in stimulated seawater purchased from J&K Scientific Ltd. Beijing, China. The deionized water (DI) used in the experiment was taken from a water purification system (B20-WTZ, Sichuan Chunjie Technology Co., Ltd., Sichuan, China). All materials were utilized without any further modification.

### 2.2. Facile Fabrication of CC@PU

**Figure 2:**
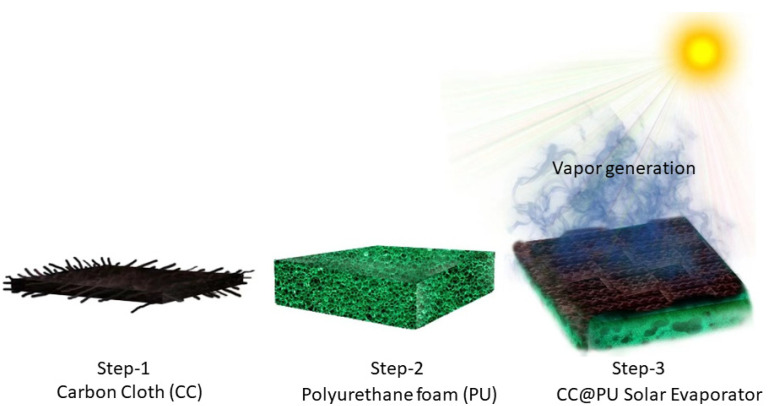
Schematic illustration of the facile fabrication process of carbon-cloth-wrapped polyurethane-based solar evaporator.

Compared to other complex structures for solar evaporation systems [22,23], the devised solar evaporator was composed of commercially available carbon cloth without any modification as a washable photothermal layer and hydrophilic polyurethane substrate for the convective flow of continuous water for better thermal management and to provide support to the photothermal layer. The selected geometry of the carbon cloth (3 × 3 cm^2^) was wrapped over the same dimensions of polyurethane foam for effective water transportation and intensified heat accumulation over the liquid/air interface. Afterward, the developed system was placed in a petri dish which was filled with stimulated seawater (3.5 wt%, NaCl). Eventually, the facile solar evaporation design was ready to examine the solar-driven seawater desalination as shown in Figure 2.

### 2.3. Controlled Solar Evaporation Experiment

The controlled solar-driven seawater evaporation investigations were carried out using a solar simulator (model PLS-FX300HU, Beijing Perfect light Technology Co., Ltd. Beijing, P.R. China) which is equipped with multiple controlled solar intensities. The optical filter was utilized to obtain a 1.5 G AM spectrum. The developed CC@PU evaporation system was functionalized in the presence of a stimulated seawater (3.5 wt%)-filled petri dish placed over an electronic analytical balance (Mettler Toledo, ME204, Singapore) with a resolution of 0.001 g which was kept under the spot solar irradiance (1 kW m^−2^ or 1 sun). The mass change of stimulated seawater was measured instantaneously, and the data were recorded after 30 min of continuous solar irradiation to obtain the actual solar-to-vapor conversion efficiency. The interfacial surface temperatures of both the CC@PU photothermal layer and water body were measured using KEYSIGHT (34972A, Tekmark Group of Companies, Kuala Lumpur, Malaysia employing two temperature sensing thermo-couples mounted on the photothermal surface and bottom surface, respectively. A thermal infrared image camera (FLIR E4 Pro, Wuhan Guide Sensmart Tech Co., Ltd. Wuhan, P.R. China) was also utilized to sense the intensified solitary heat accumulation. For the purity check of condensed water and concentration count in stimulated seawater, Inductively Coupled Plasma-Optical Emission Spectrometry (ICP-OES, EP Optimal 8000, Perkin Elmer, San Jose, CA, USA) was employed to match purity with the WHO drinking water standard. The whole experimental process was carried out under ambient conditions, at a temperature of ~25.03 °C and humidity of ~45%.

### 2.4. Photothermal Conversion Efficiency

The photothermal conversion efficiency of the developed systems was calculated by the following Equation (1) [24]:

(1) $$\eta =\frac{\left(H_{v}+H_{s}\right)\times v}{Q_{s}}$$

where η is the photothermal efficiency of the system, v is the evaporation rate (kg/m^2^ h) of water, *Hv* is the heat of vaporization of water (~2260 kJ/kg), *Hs* is the sensible heat of water, and *Qs* is the incidence solar light intensity (1.0 kW/m^2^). Where sensible heat can be calculated by the following formula:

(2) $$H_{s}=mc\Delta T$$

In the above equation, *m* is the mass of evaporated water, *c* is the specific heat capacity of water (4182 J/kg °C), and Δ*T* is the temperature difference between the initial (*T_i_*) and final (*T_f_*) temperature of water (*T_f_ − T_i_* = 67.37 °C − 25.03 °C = 42.34 °C).

## 3. Results

### 3.1. Microstructural and Morphological Analysis

**Figure 3:**
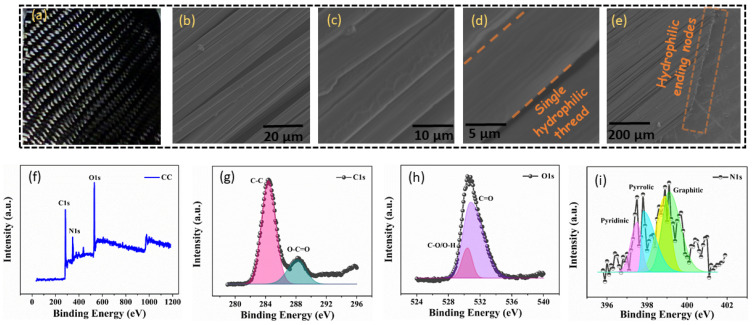
Morphological and chemical composition of black carbon cloth (CC). (**a**) Digital photo of CC. (**b**–**e**) FESEM images of the surface view of CC along with side-view nodes with different magnifications. Chemical composition of CC (**f**) full scan of XPS spectrum of CC along with elemental spectra (**g**) C1s (**h**) O1s (**i**) N1s spectra.

The self-regenerating CC@PU-based interfacial solar evaporator is fabricated by a facile bilayer structure. As a typical carbon-based composite material, CC is substantially exploited in the fields of celestial navigation, electricity generation, fire control systems, solar cells, the construction industry, and sports equipment owing to its exceptional features flexibility, excellent optical absorption, corrosion resistance, ultrahigh strength properties, and heat resistance [20]. The interfacial photothermal layer of highly solar-absorbing CC on the top of the PU foam shows an excellent solar-thermal conversion response with the hydrophilic network of carbon fiber (threads). The morphology and porous structure of the CC were examined by FESEM spectroscopy. Figure 3a shows the digital photograph of the carbon cloth, manifesting the pitch-dark surface which facilitates enhanced solar absorption. Figure 3b shows the corresponding FESEM image of the CC, wherein multiple smooth hydrophilic cylindrical fibers overlap with each other, developing numerous micro-channels in the CC. Figure 3c,d shows the high-resolution image of the CC, revealing the densely inter-weaved carbon fibers of CC (6–7 μm in diameter) which can potentially regulate the salt-rejection by self-dissolving property and facilitates easy vapor escape. Figure 3e shows the FESEM image of the abundant hydrophilic ending of CC, which provides instantaneous wettability via capillary act and ensures quick water availability from one end to the other.

The chemical composition of the CC was analyzed by performing X-ray photoelectron (XPS) spectroscopy and the obtained pattern is revealed in Figure 3f. The XPS spectra consist of three major peaks revealing the presence of C1s, N1s, and O1s at the corresponding binding energy values of 283.61, 347.20, and 530.75 eV, respectively. The fitting peaks of the C1s from the XPS spectrum are shown in Figure 3g, which exhibits the deconvolution of the C1s spectrum into two peaks attributed to the presence of the chemical bonds C–C (284.39 eV) and O–C=O (288.18 eV), respectively. The O1s spectrum of the CC is shown in Figure 3h; it was resolved into two sub-peaks at 530.42 eV and 530.82 eV binding energy values manifesting the presence of C–O/O–H and C=O chemical bonds. The peak fitting of the N1s spectrum was done by resolving it into four sub-peaks corresponding to pyridinic, pyrrolic, and graphitic nitrogen content at the corresponding binding energy values of 397.50, 397.81, 398.90, and 399.10 eV, respectively (Figure 3i). The pyridinic –N and graphitic–N is sp^2^ hybridized whereas, pyrrolic –N is sp^3^ hybridized. The pyridinic –N bonds with two C atoms at the edges and contributes one p electron to the p system [25]. Pyrrolic –N refers to N atoms that contribute two p electrons to the p system of C atoms in CC.

### 3.2. Solar Absorption & Intensified Photothermal Heat

**Figure 4:**
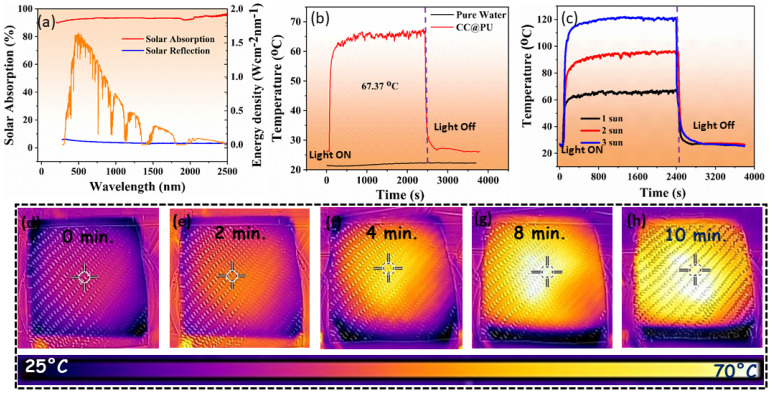
(**a**) Solar absorption and reflection spectra of carbon cloth. (**b**) Intensified photothermal heat accumulation at liquid/air interface via surface temperature spectrum of water and CC@PU. (**c**) Intensified photothermal heat via CC@PU solar evaporator over different solar intensities. (**d**–**h**) Real-time solitary heat accumulation captured via IR camera satisfied with actual surface temperature spectra.

In order to develop any solar-driven desalination device, solar absorption and photothermal conversion efficiency are vital features that play a critical role in increasing the efficiency of an evaporating device [6,13,26]. To assess the solar absorption of the CC, ultraviolet-visible-near-infrared (UV-vis-NIR) spectroscopy was performed across the entire light range of 200–2500 nm. Surprisingly, the pitch-dark surface of the CC had 92% solar absorption with a minimum reflection as shown the Figure 4a. The results affirm that CC shows distinct broadband solar absorption with overlap in the solar spectrum and efficiently captures the incident light beams within the densely overlapped carbon fibers via light scattering and multiple internal reflections and absorption. This leads toward enhanced solar absorption with an enhanced energy conversion potential and paves the path for interfacial heat localization. This exceptional absorbing potential leads to an enhancement of the incident surface temperature. For this, the two systems were developed (CC@PU and water) and placed under simulated one-solar intensity to record the comparative surface temperature enhancement as a function of the irradiation time by a thermocouple sensor probe. The obtained results were dawn in the form of graphs as shown in Figure 4b.

Upon light irradiation, the surface temperature of the CC@PU reached the equilibrium state in short periods of time, and a rapid temperature in the top increase was observed. After 1 h illumination, the highly absorbing CC@PU-based interfacial solar evaporator remarkably achieved a surface temperature enhancement up to 67.37 °C which is much higher than water and other previously reported solar-driven steam generators. Figure 4c shows the surface temperature enhancement of CC@PU under multiple solar intensities (1, 2, 3 kW m^−2^) and reveals an interfacial layer temperature enhancement up to (119.18 °C) under three solar intensities showing excellent heat accumulation and perfect thermal insulation from the downward heat conduction. This remarkable interfacial heat localization provides its potential as a promising solar-driven evaporation structure as well as heat accumulation applications. The real-time solitary heat accumulation was captured via IR camera, as revealed in Figure 4d–h. The CC@PU surface achieved intensified surface temperature in 10 min of solar irradiance (1 kW m^−2^) satisfied with actual surface temperature spectra (67.37 °C). This enhanced interfacial heat accumulation might be of great interest to future solar-driven applications especially hyperthermia-inspired disinfection, and thermal storage applications.

### 3.3. Solar Evaporation Efficiency

**Figure 5:**
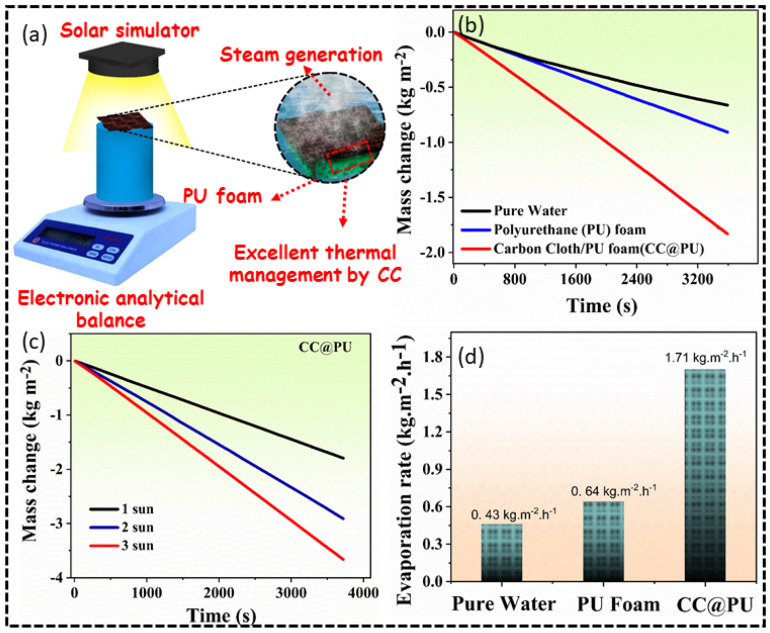
(**a**) Schematic illustration of the solar steam generation setup. (**b**) Time-dependent mass change of the pure water, PU foam, and CC@PU under 1 kW m^−2^. (**c**) Time-dependent mass change of CC@PU under 1, 2, 3 kW m^−2^. (**d**) Evaporation rates of pure water, PU foam, and CC@PU under one solar illumination.

The distinguished solar harvesting potential is specifically ascribed to the highly carbonaceous carbon cloth consisting of intertwined carbon fibers which efficiently captures the incident solar light and localizes it on the top surface via multiple reflections and absorption within the matrix, while simultaneously restricting the thermal conduction towards lower structure for effective thermal management, which forms the basis for the interfacial solar heating [27,28,29]. The hydrophilic support (PU foam) ensures efficient water transportation with the combined hydrophilicity of the CC which facilitates quick vapor escape. The evaporation rates of pure water, PU, and CC@PU were measured using a solar simulator enable to provide multiple solar intensities. Figure 5a shows the schematic illustration of the steam generation setup to measure the time-dependent mass change for 1 h for the developed three evaporation systems (pure water, PU, CC@PU) under 1 kW m^−2^ intensity using an advanced electronic analytical balance with a precision of 0.0001 g. The evaporation devices were placed on the analytical balance under the beam spot of the solar simulator (1 kW m^−2^) and the whole system was allowed to stabilize for half of an hour. Afterward, all the devices were irradiated for 1 h and corresponding time-dependent mass change was recorded via analytical balance; the obtained results are shown in Figure 5b. As obvious from the graphs, the maximum mass change upon 1 hr radiation was obtained by the highly absorbing interfacial CC@PU evaporation device as compared to pure water and simple PU foam, revealing CC@PU as a promising candidate for enhanced freshwater yield in remote areas. Moreover, the CC@PU was also irradiated by the multiple simulated solar intensities and the corresponding mass change rate was measured, which is shown in Figure 5c.

The time-dependent mass change under 1, 2, and 3 kW m^−2^ is shown in Figure 4c, showing a high mass change (3.66 kg m^−2^) under three solar irradiations, which is highly attributed to the distinctive solar capturing of CC and excellent heat localized on the top surface, the good hydrophilicity of evaporation layer, and the combined excellent thermal management of the CC and PU which ultimately facilitated in achieving the high mass change rate and eventually enhanced the evaporation efficiency. The corresponding evaporation rates of the three developed evaporation systems were calculated and drawn in the form of a bar column, as shown in Figure 5d. The maximum evaporation rate was calculated for the CC@PU which is up to 1.71 kg m^−2^ h^−1^ excluding dark conditions, which is comparatively much higher than PU foam (0.64 kg m^−2^ h^−1^) and pure water (0.43 kg m^−2^ h^−1^). This attributes the prepared interfacial CC@PU evaporation device as a renewable-energy-derived reliable device with economic and infrastructure feasibility for practical installment at the industrial level to eradicate the water scarcity issues.

### 3.4. Salt-Rejection Behavior

**Figure 6:**
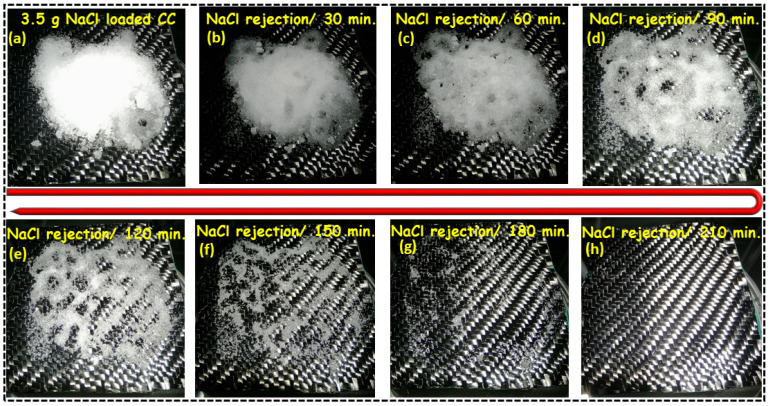
The salt-rejection experiment of CC@PU-based interfacial solar evaporation system. (**a**–**h**) Time-dependent thermal dissolution of the 3.5 g solid NaCl by interfacial CC@PU placed on the top surface under simulated seawater (3.5 wt% NaCl) was completely dissolved back to the simulated water during continuous evaporation for 210 min upon continuous solar irradiation of 1 kW m^−2^.

Many promising solutions are offered to cope with this issue, i.e., drilling millimeter channels in the wooden substrate and thin film evaporating structures, which sacrifice the photothermal area and lead to thermal leakage from the photothermal layer to the bulk water and suppresses the performance of the device [3,30]. Therefore, the self-regenerating potential of the as-prepared CC@PU interfacial solar evaporator was also checked against salt rejection in minimum record time to assess its reliability under extreme (high salt concentration) conditions, as shown in Figure 6a–h. For this, the CC@PU interfacial solar evaporation was placed in a 3.5 wt% NaCl solution and placed under 1 kW m^−2^ simulated intensity of solar simulator for several hours with an excess 3.5 g NaCl (enough to saturate the whole structure) and was spread on the interfacial photothermal layer to inspect the operational potential under extreme salt environment, as shown in Figure 6a.

Furthermore, the CC@PU interfacial evaporation device successfully dissolved the 3.5 g of solid NaCl into the underlying water upon the 210 min of constant irradiation, showing the tremendous self-generating potential of the CC@PU solar evaporator under smooth evaporation conditions. The hydrophilic carbon cloth efficiently localized the thermal energy on the top matrix which converted the water molecules into vapors approaching the air–water interface and were transported by downward hydrophilic support (PU). The generated water vapors dissolved within the salt placed on the top layer and gradually formed a concentrated brine solution. This hot brine migrated from the top surface through the hydrophilic structure of CC@PU towards the underlying bulk water via a salinity gradient. The accumulation of salt was prevented by the highly porous network of overall structure which potentially promoted the super wettability for quick water transport and vapor escape [3]. The concentration difference between the two sides pushed the salt ions from the high to lower concentration side and eventually the migration of hot brine effectively dissolved all salt content downward with no surface etching or deformation of the interfacial layer manifesting the tremendous potential of the CC@PU solar evaporator for salt rejection with no salt accumulation and interfacial surface degradation, as shown in Figure 6b–h. Hence, from the above experiments, the prepared CC@PU device was validated for supreme salt rejection with self-regenerating potential with extreme stability endorsing its potential for practical applicability for seawater desalination.

### 3.5. Purity & Long-Term Efficacy

**Figure 7:**
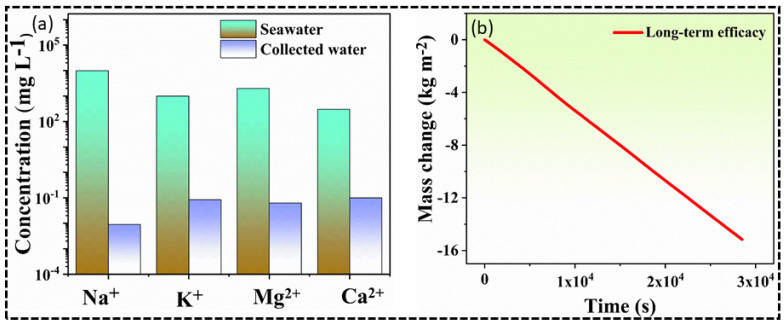
Purity standard of condensed water obtained via CC@PU solar evaporator, and long-term efficacy without any surface deterioration. (**a**) ICP-OES examination of stimulated seawater and condensed water. (**b**) Long-term operation of CC@PU solar evaporator under 1 kW m^−2^ using stimulated seawater conditions.

It is obvious that seawater contains a higher concentration of dissolved salts (3.5 wt%). Solar-driven seawater desalination is of great importance to desalinate seawater without electrical or mechanical energy [11]. Of note, many solar evaporators have been developed to desalinate seawater efficiently, unfortunately, all the parameters have not been achieved yet in a single solar evaporation structure [3]. The facile-fabricated CC@PU solar evaporator exhibited efficient solar-to-vapor conversion efficiency and simultaneously rejects salt for effective and long-term freshwater generation. The purity check of condensed water and stimulated seawater (3.5 wt%) was examined via the Inductively Coupled Plasma Optical Emission Spectroscopy (ICP-OES), as demonstrated in Figure 7a. CC@PU solar evaporator has a high potential to efficiently purify saline water. According to ICP-OES examination, condensed water has a lower concentration of salt ions especially Na^+^, K^+^, Ca^2+^, and Mg^2+^ which are essential to obtain standard drinking water as compared to higher concentrated stimulated seawater. The purified water by CC@PU solar evaporator perfectly rejects the elevated ions concentration level and the quality of the produced fresh water perfectly meets the standard of drinking water set by the World Health Organization (WHO) [11].

**Figure 8:**
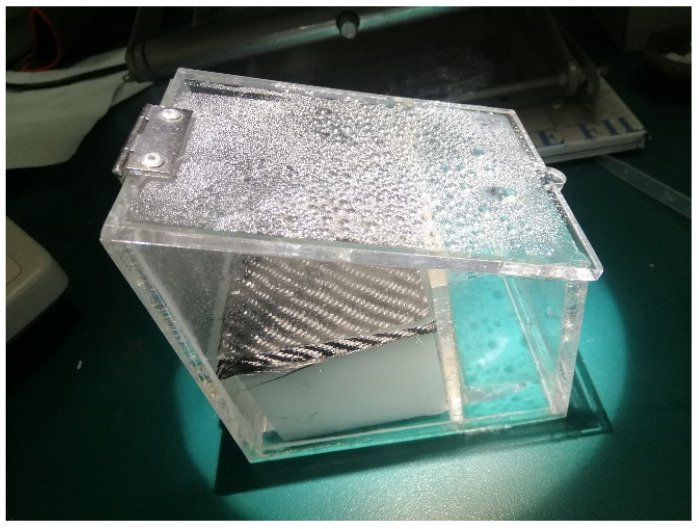
Slope-shaped glass condenser for vapor condensation.

Figure 7b represents the long-term solar evaporation test via CC@PU solar evaporator over a consecutive 8 hrs under 1 kW m^−2^ solar irradiation. Of note, the long-term evaporation experiments possessed excellent mass change (15.24 kg m^−2^ during 8 h operation) with minimum discrepancy under seawater conditions under 1 kW m^−2^ solar irradiance. There was no obvious deterioration or surface fouling observed during long-term operation. As illustrated in Figure 8, a smooth evaporation behavior was obtained and vapors were collected through a slope-shaped glass condenser which consisted of two chambers for stimulated seawater and condensed water, respectively, showing the excellent reliability of our device. Hence, the prepared device can potentially be installed at the industrial level for real-world applications. The detailed comparison of evaporation performances along with existing solar evaporators is described in Table 1**.**

Eventually, ample improvements are required to develop an all-weather solar water evaporation system including all parameters in a single evaporator such as solar absorption, mechanical robustness, salt-resistance, and higher evaporation rates. Of note, smooth vapor collection also entails low-cost condensers with excellent fogging capabilities, e.g., transparent glass or flexible polymer condensers. From Table 1, we see that the CC@PU solar evaporator exhibits a wide range of solar absorption (92%), and a higher evaporation rate (1.71 kg m^−2^ h^−1^) along with excellent solar-to-thermal conversion efficiency (90%) as compared to other successful solar evaporators [14,34,35,36,37,38,39]. The current study promotes the facile fabrication of solar evaporators without any complex infrastructure which requires only commercially available cheap materials, and higher feasibility to installation in remote-sensing areas in under-developing countries.

## 4. Conclusions

In conclusion, we successfully developed an interfacial photothermal heat accumulation-inspired solar evaporator via a facile fabricating technique derived from commercially available carbon-cloth-wrapped over a polyurethane sponge (CC@PU) for salt-resistant and freshwater generation. The state-of-the-art investigations were carried out to validate the enhanced photothermal conversion efficiency of carbon cloth, e.g., the pyridinic –N and graphitic –N is sp^2^ hybridized, whereas pyrrolic –N is sp^3^ hybridized, and the pyridinic–N bonds with two C atoms at the edges and contributing one p electron to the p system promoted enhanced solar absorption of CC (92%). The solar evaporator delivered outstanding photo-thermal conversion efficiency (90%) with a high evaporation rate of up to 1.71 kg m^−2^ h^−1^. The interfacial layer of pitch-black CC achieved enormous heat localization (67.37 °C) under 1 kW m^−2^. More importantly, the long-term evaporation experiments with minimum discrepancy under seawater conditions (mass change of water 15.24 kg m^−2^ for 8 h operation under 1 kW m^−2^ solar irradiations) inculcated the operational sustainability for multi-media rejection and self-dissolving efficacy (solid NaCl, 3.5 g/210 min). Finally, this facile fabrication showcased its potential for enhanced heat accumulation structures for freshwater production with simultaneous salt rejection for industrial applications.

## Figures and Tables

**Table 1:** Represents the detailed comparison of existing solar evaporators along with our fabricated CC@PU solar evaporator.

Sr. No	Solar Driven System	Mass Change (kg m^−2^)	Evaporation Rate (kg m^−2^ h^−1^)	Efficiency (%)	Absorption (%)	Ref.
**1.**	rGO loaded non-woven fabric	~1.6	1.37	50.4	93	[7]
**2.**	Modified cotton fabric (MCF)	~1.55	1.49	91.72	95	[31]
**3.**	Wood/PPy	1.2	1.014	72.5	99	[32]
**4.**	Candle Soot deposited Cotton fabric	~1.2	1.375	86.3	95	[33]
**5.**	HN/NiO	1.38	1.37	85.8	92.1	[14]
**6.**	Carbon foam	0.8	1.57	86	96.19	[34]
**7.**	Graphene membrane	1.4	1.37	90	99.9	[35]
**8.**	Silk/rGO	0.8	1.48	102	94	[36]
**9.**	rGo/Cotton	1.5	1.47	NA	95	[37]
**10.**	Black sand	1.2	1.43	81	98.25	[38]
**11.**	Polymer foams	1.5	1.574	90.4	94	[39]
**12.**	CC@PU	1.80	1.71	90	92	This work

## Data Availability

Not applicable.
